# Clinicians’ Perspectives After Implementation of the Serious Illness Care Program: A Qualitative Study

**DOI:** 10.1001/jamanetworkopen.2021.21517

**Published:** 2021-08-18

**Authors:** Andrew Lagrotteria, Marilyn Swinton, Jessica Simon, Seema King, Gwenn Boryski, Irene Wai Yan Ma, Fiona Dunne, Japteg Singh, Rachelle E. Bernacki, John J. You

**Affiliations:** 1 Temerty Faculty of Medicine, University of Toronto, Toronto, Ontario, Canada; 2 Department of Health Research Methods, Evidence, and Impact, Faculty of Health Sciences, McMaster University, Hamilton, Ontario, Canada; 3 Department of Oncology, University of Calgary, Calgary, Alberta, Canada; 4 Department of Community Health Sciences, University of Calgary, Calgary, Alberta, Canada; 5 Department of Medicine, University of Calgary, Calgary, Alberta, Canada; 6 iCAN ACP Research Program, Calgary, Alberta, Canada; 7 Department of Psychosocial Oncology and Palliative Care, Dana Farber Cancer Institute, Harvard Medical School, Boston, Massachusetts; 8 Division of General Internal and Hospitalist Medicine, Department of Medicine, Trillium Health Partners, Credit Valley Hospital, Mississauga, Ontario, Canada

## Abstract

**Importance:**

Discussions about goals of care with patients who are seriously ill typically occur infrequently and late in the illness trajectory, are of low quality, and focus narrowly on the patient’s resuscitation preferences (ie, code status), risking provision of care that is inconsistent with patients’ values. The Serious Illness Care Program (SICP) is a multifaceted communication intervention that builds capacity for clinicians to have earlier, more frequent, and more person-centered conversations.

**Objective:**

To explore clinicians’ experiences with the SICP 1 year after implementation.

**Design, Setting, and Participants:**

This qualitative study was conducted at 2 tertiary care hospitals in Canada. The SICP was implemented at Hamilton General Hospital (Hamilton, Ontario) from March 1, 2017, to January 19, 2018, and at Foothills Medical Centre (Calgary, Alberta) from March 1, 2018, to December 31, 2020. A total of 45 clinicians were invited to participate in the study, and 23 clinicians (51.1%) were enrolled and interviewed. Semistructured interviews of clinicians were conducted between August 2018 and May 2019. Content analysis was used to evaluate information obtained from these interviews between May 2019 and May 2020.

**Exposures:**

The SICP includes clinician training, communication tools, and processes for system change.

**Main Outcomes and Measures:**

Clinicians’ experiences with and perceptions of the SICP.

**Results:**

Among 23 clinicians interviewed, 15 (65.2%) were women. The mean (SD) number of years in practice was 14.6 (9.1) at the Hamilton site and 12.0 (6.9) at the Calgary site. Participants included 19 general internists, 3 nurse practitioners, and 1 social worker. The 3 main themes were the ways in which the SICP (1) supported changes in clinician behavior, (2) shifted the focus of goals-of-care conversations beyond discussion of code status, and (3) influenced clinicians personally and professionally. Changes in clinician behavior were supported by having a unit champion, interprofessional engagement, access to copies of the Serious Illness Conversation Guide, and documentation in the electronic medical record. Elements of the program, especially the Serious Illness Conversation Guide, shifted the focus of goals-of-care conversations beyond discussion of code status and influenced clinicians on personal and professional levels. Concerns with the program included finding time to have conversations, building transient relationships, and limiting conversation fluidity.

**Conclusions and Relevance:**

In this qualitative study, hospital clinicians described components of the SICP as supporting changes in their behavior and facilitating meaningful patient interactions that shifted the focus of goals-of-care conversations beyond discussion of code status. The perceived benefits of SICP implementation stimulated uptake within the medical units. These findings suggest that the SICP may prompt hospital culture changes in goals-of-care dialogue with patients and the care of hospitalized patients with serious illness.

## Key Points

**Question:**

What were hospital clinicians’ experiences and perceptions of the Serious Illness Care Program (SICP), a multifaceted capacity-building intervention to improve communication with patients who are seriously ill?

**Findings:**

In this qualitative study including 23 clinicians from 2 hospitals in Canada, clinicians stated that the various structural and organizational elements of the SICP supported changes in their behavior, shifted the focus of goals-of-care conversations beyond discussion of resuscitation preferences (ie, code status), and influenced them on personal and professional levels.

**Meaning:**

The findings of this study suggest that clinicians may find the SICP helpful in supporting them to engage in meaningful communication that goes beyond discussion of code status with patients who are hospitalized with serious illness.

## Teaser

This qualitative study explores clinicians’ experiences with the Serious Illness Care Program 1 year after its implementation in 2 hospital wards in Canada.

## Introduction

Hospitalized patients with serious life-limiting illness often receive treatment incongruent with their preferences.^1,2,3^ Unwanted interventions are associated with increased distress among patients and families, medical error, reduced quality of life, and lower satisfaction with care.^4,5,6^ Clinicians experience moral distress when providing aggressive interventions for which they perceive no benefit.^7^ Person-centered conversations focused on patients’ illness understanding, goals, fears, sources of strength, values, and acceptable tradeoffs can support more goal-consistent care.^8^ However, such discussions are typically infrequent, occur late in the illness trajectory, and generally focus more on patients’ resuscitation preferences (ie, code status) rather than their values and goals.^1,2,3,9,10,11,12,13,14,15^

Multiple educational programs and tools have sought to enhance this communication.^13,16,17,18^ The Serious Illness Care Program (SICP) uses a multifaceted system-change approach to build clinician capacity for more timely and person-centered conversations about the values and priorities of patients with serious illness.^19^ In a cluster randomized clinical trial, the SICP achieved more, earlier, and better-quality conversations in an outpatient setting and reduced anxiety and depression among patients with advanced cancer.^20,21^ However, less is known about the consequences of the SICP when applied in hospitals. A systematic review of advance care planning for hospitalized older adults with frailty also concluded that physician experiences have not been well explored.^22^ The objective of this qualitative study was to understand clinicians’ experiences with the SICP 1 year after its implementation in the general internal medicine wards of 2 hospitals in Canada.

## Methods

### Design

We used qualitative description to focus on clinician experiences with the SICP.^23,24,25,26^ Qualitative description emphasizes describing participant experiences in their language with minimal interpretation and is recommended for research questions that aim to report participant experience.^23,24,25^ The study was approved by the Hamilton Integrated Research Ethics Board. All participants provided written informed consent. This study followed the Consolidated Criteria for Reporting Qualitative Research (COREQ) reporting guideline for qualitative studies.^27^

### The SICP Intervention

The SICP is a multifaceted capacity-building communication intervention. Its principal component, the Serious Illness Conversation Guide (hereinafter referred to as the guide), provides evidence-based questions and a conversation framework to explore, with topics including illness understanding, prognosis, values, goals, fears, sources of strength, essential abilities, acceptable tradeoffs, and family understanding (eMethods 1 in the Supplement).^19^ Other program elements include a patient preparation letter, a postconversation family communication guide, and a 2.5-hour interactive clinician training session. System-change components include routine identification of patients at high risk of death, a method to prompt clinicians to initiate the conversation, and a structured template for documentation.^19^

### Setting and Participants

This study was conducted in the general internal medicine wards of 2 Canadian teaching hospitals (Hamilton General Hospital, Hamilton, Ontario, and Foothills Medical Centre, Calgary, Alberta) (Table 1). The SICP was implemented at Hamilton General Hospital from March 1, 2017, to January 19, 2018, and at Foothills Medical Centre from March 1, 2018, to December 31, 2020. At both sites, a unit nurse who was seconded to the study (ie, temporarily assigned to assist with the study) supported implementation by identifying and preparing patients, prompting clinicians to have conversations, and scheduling conversations. Of 45 clinicians invited to participate, 23 clinicians (51.1%; 11 from the Hamilton site and 12 from the Calgary site) were enrolled and interviewed. Semistructured interviews of clinicians were conducted between August 2018 and May 2019. Content analysis was used to evaluate information obtained from these interviews between May 2019 and May 2020 (1 year after implementation of the SICP).

**Table 1.** Characteristics of Participating Hospitals and Implementation Teams

Characteristic	No.	
	Hamilton General Hospital	Foothills Medical Centre
Location	Hamilton, Ontario	Calgary, Alberta
Medical wards	3	1
Patient beds	100	28
Attending physicians in ward at a given time	6	3
Clinicians at site	28	29
Physicians	21	29
Nurse practitioners	4	0
Social workers	3	0
Clinicians approached for participation	16	29
Physicians	12	29
Nurse practitioners	3	0
Social workers	1	0
Members of implementation team	19	15
Meetings per year during planning and implementation phase	6	6
Background of implementation team		
Clinical staff	Yes	Yes
Nonclinical staff	Yes	Yes
Hospital administrative staff (eg, unit manager)	No	Yes
External stakeholder (eg, external to general medical ward)	Yes	Yes
Patient advisors	Yes	Yes

### Sampling and Data Collection

The Hamilton site used purposive sampling to intentionally include diverse clinician perspectives (ie, physicians, nurse practitioners, and social workers), whereas the Calgary site invited all physicians to participate. Clinicians received an email from the site’s primary investigator inviting them to participate. Interviews were conducted individually using a semistructured guide and were 30 to 45 minutes in duration (eMethods 2 in the Supplement). Interviewers (A.L. and M.S.) were not known to the participants and did not ask participants to provide feedback on the findings. Clinicians at the Calgary site were interviewed via video conference or teleconference.

### Data Analysis

Interviews were digitally recorded, transcribed, and deidentified, and analysis was performed from May 2019 to May 2020. Conventional content analysis consistent with the qualitative descriptive approach was used to analyze transcripts.^28,29^ Independent line-by-line open coding of 5 transcripts was conducted (A.L. and M.S.), and a preliminary list of codes was developed through consensus during a series of meetings. The lead analyst (A.L.) coded the remaining transcripts. Coding reports from the entire data set and 4 transcripts were reviewed during 3 meetings with the analysis team; data saturation was confirmed. New insights identified during these meetings were incorporated into the analysis by the lead analyst; all decisions and coding revisions were documented in an audit trail.^30^ Associations between codes were discussed at these meetings and were used to inform the organization of codes into meaningful categories and higher-level clusters.^31^ The research team comprised a group of clinicians, nonclinician qualitative researchers, and a patient advisor. The interviewers had no relationship with the participants. Data were managed and analyzed using NVivo software, version 12 (QSR International).

## Results

Among 23 clinicians enrolled and interviewed, 15 participants (65.2%) were female (Table 2). The mean (SD) number of years in practice was 14.6 (9.1) at the Hamilton site and 12.0 (6.9) at the Calgary site. Participants included 19 general internists, 3 nurse practitioners, and 1 social worker.

**Table 2.** Participant Characteristics

Characteristic	No. (%)	
	Hamilton General Hospital	Foothills Medical Centre
Total participants, No.	11	12
Type of clinician		
General internist^a^	7 (63.6)	12 (100)
Nurse practitioner	3 (27.3)	0
Social worker	1 (9.1)	0
Sex		
Male	3 (27.3)	5 (41.7)
Female	8 (72.7)	7 (58.3)
Time in practice, mean (SD), y	14.6 (9.1)	12.0 (6.9)
Scheduled SICP conversations, mean (SD)	12.6 (6.3)	9.6 (10.1)
Scheduled SICP conversations per week while participating in study, mean (SD)	1.1 (0.5)	0.8 (0.8)

Abbreviation: SICP, Serious Illness Care Program.

^a^ All internists were attending physicians.

The 3 main themes were the ways in which elements of the SICP implementation (1) supported changes in clinician behavior, (2) shifted the focus of goals-of-care conversations with hospitalized patients beyond discussion of code status, and (3) influenced clinicians personally and professionally.

### Elements Supporting Changes in Clinician Behavior

Clinicians said that several elements of the program supported behavior change: (1) having a unit champion present, (2) engaging interprofessional team members, (3) having copies of the guide accessible, and (4) documenting conversations about serious illness using a template in the electronic medical record. Together, these elements of the intervention were described as providing structure. As 1 clinician described, “it sort of legitimizes this [having serious illness conversations] as something that’s very important for physicians to do.”

#### Having a Unit Champion Present

Many described the presence of a unit champion as one of the most important program elements. One clinician said, “There’s a cueing reminder, there’s an administrative burden removed where someone else is scheduling and letting me know when it [the conversation] is, based on my availability, and that really helps. The patients and family are primed on it. It was just any time I wanted to do it, and whenever we planned to do it, it just happened. Everyone was on board, and everything was set up.”

Many clinicians expressed opinions that were similar to 1 clinician’s statement that “the biggest challenge is sort of creating a formal time or space to have these conversations.” However, the unit champion’s coordination of a scheduled time to meet helped in overcoming this challenge: “I think the nudging from…[the unit] champion, supporting the program, is probably the thing that’s facilitated the most in terms of actually using the guide, of getting over that hurdle to make the time to do it.”

#### Engaging Interprofessional Team Members

Many clinicians described the ways in which the program empowered nonphysician members of the interprofessional team, especially with patient identification. As 1 clinician stated, “It was the bedside nurse that said, ‘You’ve made this care plan, but whenever I go in there, he keeps refusing things. Can you please check and make sure this is what he wants?’…I feel like without this program, she might not have felt empowered to voice it.”

A similar experience was shared by a nurse practitioner, who said, “I also feel like the whole team has embraced this process from all the varied allied health professions. Everyone is very aware of the process and so, when someone brings it up, it’s like, ‘Yeah, you’re right. That would be appropriate.’ I think we’re all so invested in this process because we know it helps and it helps to give better care to our patients.”

One clinician recognized improvements; however, the clinician was uncertain about the factors to which that success should be attributed, stating, “I think we’re doing a better job of identifying these patients earlier, and whether it’s attributable to this program, specifically, or this is just supporting a culture shift in that regard, I’m not very sure.”

#### Having Copies of the Guide Accessible

When asked about which elements of the program facilitated participation, many clinicians referred to having copies of the guide accessible. One clinician responded, “Having copies on the ward. There’s a place that all the materials are kept on our unit, and they’re laminated guides. So, I would take one of those with me every time…to refresh my memory as to how the conversation unfolds and the specific phrasing of the questions.” Another clinician responded, “The sheets with the conversation guide that you can take off the wall and take into the patient's room with you. You can just take it and do it, like, right there. So, that makes it really easy to use.”

#### Documenting Serious Illness Conversations

The inclusion of a formal documentation step within the program was described as bringing more attention to the conversation among the medical team and facilitating care over time that was more consistent with patient wishes. “When we did our training, we were asked to document the conversation in our electronic medical record under a certain document…I have found that very, very helpful,” a clinician said. Another stated, “If you're dictating in a conversation, it’s clear to everyone that this is what this person wanted, and it’s dictated, it’s easy to pull up, people readily access it. Even the dictations that I’ve dictated…I have had people readmit and they get readmitted to our team and even it’s been some time later. It’s like, okay, this is what the person wanted in this serious illness conversation at this time.”

However, the benefit of tailoring care to support patients’ wishes in the long term through the documentation process was not recognized by all. One clinician stated, “It’s going to become a highly personalized experience, but then, unfortunately, we’re on for 1 week at a time, and here I am collecting all this information and whatnot…and then I never see the patient again.”

### Elements Shifting Conversation Focus

Clinicians described 3 important elements of the program that shifted the focus of conversations: (1) changing what is asked and creating space for conversations, (2) facilitating understanding of the patient’s illness, and (3) altering clinician agendas.

#### Changing What Is Asked and Creating Space for Conversations

Many clinicians discussed the SICP’s role in changing what is asked and creating space for conversation. As 1 clinician said, “[The guide] creates a completely different environment because you’re asking questions about their bigger life values and goals…We don’t ask these things. It’s really pivotal for me; it really changes my practice.”

With regard to booking a scheduled meeting in a space other than the patient’s room, another clinician stated, “I think what I found most useful was a separation of time and space; [it] created a moment to build a relationship in a way that we don’t always in acute care because we don’t either have the time or the dynamic is different.”

#### Facilitating Understanding of Patient’s Illness

Clinicians mentioned the role of the SICP in facilitating patient and family understanding of the patient’s illness, saying that it helped to “have everyone on the same page.” One clinician stated, “I had 1 family where there were things that [the patient] expressed that they hadn’t known…it was clear that this family hadn’t gone through this before, or the kids had not appreciated their father’s concerns…they came later and really thanked me for having [the conversation].”

#### Altering Clinician Agendas

Many clinicians said that the program and guide altered their agenda for conversations, which had often previously focused on obtaining the patient’s code status. According to 1 clinician, “Before, when we would say, ‘oh, we’re going to go have a goals-of-care discussion with that patient,’ invariably, it always meant we wanted to downgrade their goals of care from, you know, full code to something less than full code.”

Another clinician also said that they “had more meetings where there really has been less of an agenda,” and using the guide helped them with “slowing down and just being a bit open-minded, not having your thoughts of a patient bound to a ‘ticky’ box…and really understanding each patient as an individual.”

Clinicians also commented on observed changes in the dialogue between care team members. As 1 clinician stated, “When someone comes in sick, the first thing you want to do is to find out what their code status is…but I’ve noticed now that people are taking a pause on that issue [and] dealing with the person as a human being. When they do discuss code status, I hear it described a bit more as…people talk about the patient’s wants, needs, or values.”

### Elements Influencing Clinicians Personally and Professionally

Personal and professional influences of the SICP that were identified by clinicians included (1) increasing comfort with having serious illness conversations, (2) bringing meaning to their work and reducing moral distress, and (3) humanizing care and tailoring a care plan to support patient wishes.

#### Increasing Comfort With Having Serious Illness Conversations

Several clinicians described the ways in which their training and use of the guide made having decision-making conversations easier. According to 1 clinician, “Now that we actually have a formal template that’s being studied, and we have training on it, I realized how patients are so willing to open up and speak, and it actually does work, some of these specific questions, of areas to explore. It allows me to be more using of it, and it’s nice using something that’s been validated, researched, so it really adds to the toolbox. I might not have asked some of those questions before, but I’m far more confident to do it now.”

Evidence of increased comfort with having conversations about serious illness was commonly found in clinicians’ descriptions of using the guide with patients “on the fly” and in other circumstances and settings. A clinician said, “I moved it across to more patients…I’ve done it in a more informal way…to explore, to some degree, even if not at complete depths, what it is the patient feels about what they’re going through and how I can help with the, especially, quality of their life.”

Although many clinicians stated that their comfort with conversations about serious illness increased because of the SICP, a few said that it could disrupt the natural flow of a conversation. One clinician said, “I just think just the static nature of it makes it a bit difficult to adapt to an actual, real-life conversation sometimes.”

#### Bringing Meaning to Work and Reducing Moral Distress

Most clinicians found that the connections they made with patients as a result of using the guide brought meaning to their work. As 1 clinician said, “It definitely…makes the day feel much more fulfilling to be able to connect with the patient or family on that deeper level as opposed to the more superficial and very busy tasks of the day.” A nurse practitioner stated, “When I do have these conversations…it makes my work very meaningful.”

A few participants described the ways in which using the guide helped them deal with moral distress, with 1 nurse practitioner saying, “even if I don’t agree [with] my challenging individual who wanted everything done, full code…I felt quite satisfied that I was respecting his wishes and that even though it wasn’t in accordance with my values, that we were doing the right thing.” Having a conversation about serious illness and “getting the patient’s input into what we were doing” was described by another clinician as relieving a “burden.”

#### Humanizing Care

By supporting clinician participation and shifting the focus of the conversation, the patient-physician interaction was perceived by many clinicians to create more humanizing care, which also allowed care to be tailored to support patients’ wishes. One physician described providing “a different type of care and really a kinder type of care,” and another clinician described having “a little more of an empathetic edge,” explaining that “when it’s so busy, it’s easy to…and I don’t use it as a bad term in a bad way…dehumanize people and process people. Just because you’re trying to get through, you become mechanical in what you say and what you do.”

One clinician said, “We explored other things that are meaningful for them right now and what would need to happen in order to be able to ensure that we maintain those wishes and look at how to help the individual feel as comfortable as possible. Like, one of my families was talking about Christmas and getting through the holidays and how to be able to have 1 more visit at home.”

## Discussion

In this qualitative study, hospital-based clinicians described the SICP as supporting changes in their behaviors and shifting the focus of conversations from intervention to values. Clinicians reported many positive influences of this shift on both personal and professional levels.

The findings are consistent with other studies of clinician experiences after using the guide. A survey study of oncologists found that the guide facilitated timely and effective conversations and increased satisfaction with their role.^32^ Primary care physicians who implemented the SICP described changes to their mindset and norms, such as an awareness of the need to prompt conversations earlier in the course of illness.^33^ The increased frequency and content of conversation documentation has been reported in other studies,^20,34^ including in a context identical to that of our study.^34^ The findings of the present study add data suggesting that hospital-based clinicians appreciated the accessibility of the guide and the clear documentation of patients’ values and goals during future encounters. In addition, the SICP’s role in bringing meaning to clinicians’ work and reducing moral distress supports the fourth facet of the quadruple aim of health care, which is improving clinician satisfaction.^35^

Clinical contexts,^36^ including staff, organization, process,^37^ and patient factors,^38^ are known to have implications for conversations about serious illness and the uptake and sustainability of practice changes. Our findings highlight that the multifaceted design of the SICP, especially the system-change components (the presence of a unit champion, the accessibility of the guide within units, and the formalized documentation procedure), supported behavior change among clinicians. Moreover, we posit that the multifaceted system changes produced greater awareness of the program within the units and was associated with the improvements in the engagement of interprofessional team members reported by our participants.^39^

Concerns with the program included finding time to have conversations, building transient relationships, and limiting conversation fluidity. However, clinicians nonetheless perceived changes in practice behaviors, suggesting that the system changes introduced by the SICP had benefits for workflow, including the cueing of practitioners and the creation of time and space for conversations.^40^ This finding emphasizes the importance of system change for successful implementation of the SICP; other studies have found that serious illness communication training alone was not associated with such behavior changes.^40,41^

### Limitations

This study has limitations. These limitations include the use of purposive sampling at the Hamilton site, whereas a representative sample was sought at the Calgary site. Both sites yielded interviews with clinicians who reported a range of experiences and frequency of conversations using the guide. Interviews were conducted in person with clinicians in Hamilton and remotely with clinicians in Calgary. This decision may have produced differences in sharing experiences; however, differences in the depth of experiences shared between sites were not detected. Furthermore, we did not interview other unit staff, such as bedside nurses, nursing managers, or unit clerks, about their perceptions of the SICP. We also recognize that any changes associated with shifting the focus of conversations are only from the perspective of the clinicians, and this theme is not informed by patient insights.

## Conclusions

The SICP was described by hospital-based clinicians as supporting changes in clinician behavior, shifting the focus of goals-of-care conversations from an emphasis on code status to patient values, and influencing clinicians on personal and professional levels. By encouraging clinician behaviors that prompt the person-centered care and emotional support valued by patients, the SICP may help to facilitate therapeutic encounters that improve the quality of care for patients with serious illness and their families.

## Notes

Supplement. **eMethods 1.** Serious Illness Care Program Interview Guide for Clinicians **eMethods 2.** Serious Illness Conversation Guide
