# Supplementary material for: Clinicians’ Perspectives After Implementation of the Serious Illness Care Program: A Qualitative Study
Source: JAMA Netw Open. 2021 Aug 18;4(8):e2121517. doi: 10.1001/jamanetworkopen.2021.21517 (PMC8374609; doi:10.1001/jamanetworkopen.2021.21517)
Supplement: Supplement. — eMethods 1. Serious Illness Care Program Interview Guide for Clinicians eMethods 2. Serious Illness Conversation Guide [file jamanetwopen-e2121517-s001.pdf]

Supplementary Online Content

Lagrotteria A, Swinton M, Simon J, et al. Clinicians' perspectives after implemention of the Serious Illness Care Program: a qualitative study. *JAMA Netw Open*. 2021;4(8):e2121517. doi:10.1001/jamanetworkopen.2021.21517

eMethods 1. Serious Illness Care Program Interview Guide for Clinicians

eMethods 2. Serious Illness Conversation Guide

This supplementary material has been provided by the authors to give readers additional information about their work.

© 2021 Lagrotteria A et al. *JAMA Network Open*.

eMethods 1. Serious Illness Care Program Interview Guide for Clinicians

Questions about Use and Influence

1. We'd like to start out by asking about your experience using the Serious Illness Conversation Guide.
    a. approximately how many times have you used the Guide with a patient or family member?
    b. can you describe anything that made it easy for you to use the Guide?
    c. was there anything that made it difficult for you to use the Guide?
2. Can you recall a time where using the Guide changed the way you interacted with a patient or their family?
    a. If yes:
        i. how did it change things?
        ii. did it change the care that was provided to this patient/family?
        iii. did it change how you felt about or understood the patient?
        iv. did you feel it changed how you felt about the care you were providing?
    b. If no, do you have any comments on why using the Guide hasn't changed the way you interact with patients and families?
3. Have your perceptions about having these conversations with seriously ill patients changed over the course of the implementation of the Serious Illness Care Program?
    a. If yes, can you describe how they have changed? (probes: re: experience with eliciting patient values, fears, trade-offs)
    b. If no, do you have any comments on why your perceptions haven't changed?
4. Has the Serious Illness Care Program had any influence on you as a clinician?
5. Have there been any changes within your clinical team in terms of how these conversations occur of how these conversations are documented?
6. Has the implementation of the Serious Illness Care Program created any change on the medical ward?
    a. has the program led to any unintended consequences on the unit?
    b. have you observed any change in the culture of the unit? (probe: changes in interprofessional communication/teamwork around these conversations, changes in awareness about the importance of these conversations)

Questions about Implementation, Sustainability & Program Expansion

1. Thinking about how the Serious Illness Care Program has been implemented on the ward, is there anything that you think could have been done differently?
2. Thinking about the 2.5 hour clinician training workshop you attended, are there any topics that you feel would have been helpful to have included in the training?
3. How sustainable to you think the SICP is for the future?
4. Do you think the program is likely to continue when the funded role for the unit champion ends?
5. What challenges are there for the program's sustainability? Can you suggest any strategies to ensure the program's sustainability?
6. If another medical unit was planning to implement the Serious Illness Care Program what advice would be important to share with them?

© 2021 Lagrotteria A et al. *JAMA Network Open*.

Closing Questions

1. Reflecting on your experience with the SICP over the last year, have there been any surprises?
2. Is there anything you would like to expand on or add to our discussion today?

© 2021 Lagrotteria A et al. *JAMA Network Open*.

eMethods 2. Serious Illness Conversation Guide

Serious Illness Conversation Guide

CLINICIAN STEPS

- ☐ **Set up**
  - Thinking in advance
  - Is this okay?
  - Combined approach
  - Benefit for patient/family
  - No decisions today
- ☐ **Guide** (right column)
- ☐ **Summarize and confirm**
- ☐ **Act**
  - Affirm commitment
  - Make recommendations to patient
  - Document conversation
  - Provide patient with Family Communication Guide

Draft R4.2 12/10/13

© 2012 Ariadne Labs: A Joint Center for Health Systems Innovation and Dana-Farber Cancer Institute

CONVERSATION GUIDE

Understanding	What is your understanding now of where you are with your illness?
Information preferences	How much information about what is likely to be ahead with your illness would you like from me?<br>FOR EXAMPLE:<br>Some patients like to know about time, others like to know what to expect, others like to know both.
Prognosis	***Share prognosis, tailored to information preferences***
Goals	If your health situation worsens, what are your most important goals?
Fears / Worries	What are your biggest fears and worries about the future with your health?
Function	What abilities are so critical to your life that you can't imagine living without them?
Trade-offs	If you become sicker, how much are you willing to go through for the possibility of gaining more time?
Family	How much does your family know about your priorities and wishes?<br>(Suggest bringing family and/or health care agent to next visit to discuss together)

© 2021 Lagrotteria A et al. *JAMA Network Open*.
